# Ex-vivo characterization of circulating colon cancer cells distinguished in stem and differentiated subset provides useful biomarker for personalized metastatic risk assessment

**DOI:** 10.1186/s12967-016-0876-y

**Published:** 2016-05-12

**Authors:** Natalia Malara, Valentina Trunzo, Umberto Foresta, Nicola Amodio, Stefania De Vitis, Laura Roveda, Mariagiovanna Fava, MariaLaura Coluccio, Roberta Macrì, Anna Di Vito, Nicola Costa, Chiara Mignogna, Domenico Britti, Ernesto Palma, Vincenzo Mollace

**Affiliations:** IRC-FSH Laboratories, Department of Health Science, University “Magna Graecia”, Viale Europa, Località Germaneto, 88100 Catanzaro, Italy; Cellular Toxicological Laboratory, Department of Health Science, Salvatore Venuta Campus, University “Magna Graecia”, 88100 Catanzaro, Italy; Medical Oncologic Department of Experimental and Clinical Medicine, Salvatore Venuta Campus and Cancer Centre of Excellence, “Magna Graecia”, 88100 Catanzaro, Italy; Bionem Laboratories, Department of Experimental and Clinical Medicine, Salvatore Venuta Campus, University “Magna Graecia”, 88100 Catanzaro, Italy; Oncologic Surgery Unit, Cancer Centre of Excellence, “Magna Graecia”, 88100 Catanzaro, Italy; Department of Experimental and Clinical Medicine, Salvatore Venuta Campus, University “Magna Graecia”, 88100 Catanzaro, Italy; Department of Health Science, Salvatore Venuta Campus, University “Magna Graecia”, 88100 Catanzaro, Italy

**Keywords:** Circulating colon tumor differentiated/stem cells, Metastatic-risk stratification

## Abstract

**Background:**

Circulating tumor cells (CTCs) represent one of the most interesting target in improving diagnosis, prognosis and treatment. Herein we evaluate the possibility of using an emo-cytometric approach on the evaluation of the heterogeneous population of CTCs to improve personalized metastatic risk assessment. We benchmarked ex vivo behavior of distinct subsets of circulating colon tumor cells with correspondent clinical behavior of patients from which we isolated CTCs.

**Methods:**

Isolation and CTC expansion were performed by a gradient protocol. In vitro characterization was determined by flow cytometry, immunofluorescence, western blotting and proteomic profiling. Cell sorter was performed with immunomagnetic beads. Confocal microscopy was used to evaluate tissue sections. Kaplan Mayer curves was cared for through Medcalc program.

**Results:**

We collected heterogeneous CTCs, derived from the whole blood of seven patients affected by colon cancer, expressing CD133^pos^CD45^neg^ (5 ± 1) and (2 ± 1) and CK20^pos^CD45^neg^ of (29 ± 3) (11 ± 1) cells/ml in Dukes D and A stage respectively. Proliferation rate of 57 ± 16 %, expression for CXCR4^pos^ of 18 ± 7 % and detectable levels of IL-6, IL-8 and SDF-1 cytokines in conditioned culture medium characterized short-time expanded–CTCs (eCTCs). ECTCs organized in tumor sphere were CD45^neg^CD133^pos^ while in adhesion were CXCR4^pos^CK20^pos^. These two subsets were separately injected in mice. The first group of xenografts developed superficial lesions within 2 weeks. In the second group, in absence of growing tumour, the survival of injected eCTCs was monitored through SDF-1 serum levels detection. The detection of human cancer cells expressing CK20, in mice tissues sections, suggested a different biological behaviour of injected eCTC-subsets: tumorigenic for the first and disseminating for the second. The benchmarking of the experimental data with the clinical course highlights that patients with prevalence of circulating cancer stem cells (CD45^neg^CD133^pos^) have a lower overall survival. Conversely, patients with prevalence of circulating differentiated cells (CXCR4^pos^CK20^pos^) have a low disease-free survival.

**Conclusion:**

On the basis of the heterogeneous composition and despite the low number of CTCs, it was possible to distinguish two subgroups of CTCs, suggesting a different clinical outcome. CTC-subsets detailing is useful to better define the metastatic–risk personalized score thus improving disease management and reducing patient care cost.

**Electronic supplementary material:**

The online version of this article (doi:10.1186/s12967-016-0876-y) contains supplementary material, which is available to authorized users.

## Background

Circulating tumor cells (CTCs) are a highly heterogeneous population of circulating progeny derived from primary tumor lesion [1]. Their circulation in the bloodstream has a bidirectional dynamism because CTCs are released and attracted by tumor tissue that transiently recaptures and releases them through the cytokines production [2]. This dynamism guarantees a continuous replacement of the CTCs pool in the bloodstream, so the characterization of these cells can detail the molecular changes occurring in the cancer lesion/s during its evolution. Up to date, the isolation and characterization of CTCs through a simple blood test represent an important step in monitoring cancer patients. In fact, only the analysis of the cancer cells referred by tissue biopsy is regarded as appropriate in the diagnostic and prognostic evaluation. But often the tissue biopsy cannot be performed for reasons related to poor clinical condition or due to critical location of the tumor or for its numerous relapsing lesions. For this reason, the liquid biopsy designed for the isolation of cancer cells constitutes a potential alternative for clinicians.

For the optimization of both CTCs isolation and their in vitro expansion procedures here described, we started from the consideration that CTCs release happens in two ways, (i) spontaneously—due to high growing tumor profile—and (ii) by active extravasations process—due to the acquisition of metastatic phenotype. Taking into account the heterotypical composition of the tumor tissue generating circulating cancer progeny [3, 4] we examined the cell populations of peripheral blood samples, without preliminary specific selection. The most used and established methodology to separate different hematological cellular populations within a blood sample, is the density gradient centrifugation [5]. In this work, by applying density gradient we highlighted the presence of CTCs of colon cancer in referred density range (working density phase). Moreover, considering that the hallmark of cancer is the higher proliferation rate, we tested CTCs cellular growth efficiency in vitro. Expanded-CTCs (e-CTCs) become viable for further molecular characterization and numerically sufficient for developing human orthotropic xenografts. Many studies have shown that the tumor tissue has a hierarchical organization. In particular, cancer cell subpopulations are arranged into tumorigenic (cancer stem cells compartment) and non-tumorigenic cells (cancer differentiated cells compartment). Relative studies distinguishing CTCs subgroups were reported [6, 7] and in particular, high count of CTCs expressing cancer stem cell phenotype is considered sign of adverse prognosis [8, 9]. In this study, we tried to distinguish staminal and differentiated CTCs in the bloodstream of colon cancer patients, to transplant these cells in immunodeficient mice for unmasking their biological properties.

## Experimental section

### Patients

All patients and healthy volunteers enrolled at University of Catanzaro (from February 2011) were informed of the investigational aspects of this study and provided written consent in accordance with local and international institutional guidelines [10]. Local ethical committee approved the study with the number: 2013.34. Peripheral blood samples collected amounted to a volume of 5 ml for each patient. However, a volume of 15 ml was collected from seven patients, five males and two females without any type of previous anticancer treatments, to develop the xenograph. The average age was 60 years, ranging from 34 to 76 years. Two independent pathologists confirmed the histological diagnosis of cancer. Stage was determined in agreement with TNM stage (Tumor-Node-Metastasis, UICC-2009) and Dukes Classification.

### Methods

#### Isolation and expansion (short-term cultivation)

Ficoll-Paque Plus (GE Healthcare) was used for cancer cells separation and the 1.080 ÷ 1.090 g/ml gradient phase [6, 11] was identified as working density phase. The working density phase of CTCs Cytokeratin 20 (CK20)^pos^ and CD45^neg^ was verified for sensitivity, specificity and purity, as reported in Additional file 1: Figure S1. Moreover, to confirm the specificity of the working density phase we performed: (a) a simulation experiment (Additional file 1: Figure S2) with a mixture of hematological cells and colon cancer cells (different concentration of GFP-conjugated HCT116 were put in entire blood sample and were evaluated through cytometric analysis); (b) we compared the working density phase with the overlaid one, 1.077–1.080 (g/ml), in which monocytes and lymphocytes (mononuclear cell layer) are located, for the presence of CK20 and Fibronectin positive cells. The final resolution of the simulation experiment was estimated of 5.8 cell/ml, as detailed in Additional file 1: Figure S2.

**Fig. 1:**
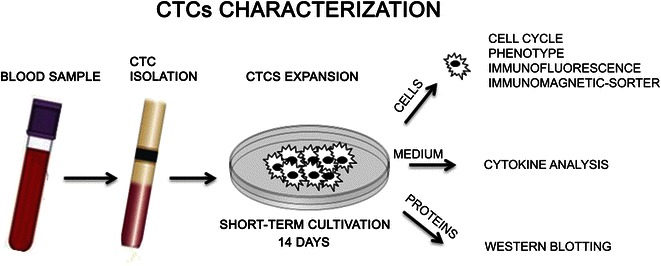
Graphic representation of applications performed on CTCs cultivated for 14 days. The short-term cultivation provided CTCs available for cytometry evaluation of cell cycle phases distribution, immunofluorescence, protein extraction and cell-sorter protocols. Moreover, conditioned medium (aged 14 days) was collected and cellular production was analyzed

After the isolation of the CTCs from the working density phase, phosphate buffered saline (PBS)-washed CTCs were recovered in a culture medium used to promote cells adhesion [6, 11, 12] and in parallel in a culture medium promoting sphere formations, as previously reported [7]. Briefly, sphere medium used was D-MEM/F12- Heparin 0.5 U/ml, EGF 50 ng/ml, FGF 25 ng/ml, BSA 1 %, penicillin–streptomycin solution 1 %. 1/3 of washed cells was processed for cytometric analysis to set up the phenotypes just before the cell seeding. CTCs were expanded for 14 days (short-term cultivation). The short-term cultivation provided (Fig. 1) amounts of CTCs sufficient for cytometry and immunofluorescence protocols. Cells were observed with Inverted Confocal Microscopy Nikon TE2000 using 408 (blue fluorescence) and 543 (red fluorescence) filters, images were digitally captured and merged using Adobe Photoshop software (v7.0).

Moreover, protein extraction was performed as previously described [11], and the conditioned medium (aged 14 days) was analyzed (Proteome Profiler, RD system).

#### Characterization of eCTCs

For flow cytometry analysis, cells were washed and incubated at 4 °C with primary antibodies. In particular, Epcam-FITC and CD133-PE (Miltenyi Biotec), CD45-APC-H7 (BD), fibronectin (SouthernBiotech) and CK20 (Abcam) were used. Moreover, secondary antibody (Southern Biotech) conjugates with FITC or PE were used. All primary antibodies were used at a dilution of 1:100 and secondary at 1:1000. Flow cytometry was performed with a FACS Canto II (Becton–Dickinson) analyzed with Cellquest software. S-phase and DNA content was carried out as previously described [13].

For CXCR4 MACS-sorting CXCR4-antibody (clone 12G5; R&D) was used and eCTCs-CXCR4^pos^ were isolated using immunomagnetic CXCR4-microbeads and a magnetic cell separation device (Midi-MACS; Miltenyi Biotec). Briefly, all cultivated cells were detached by trypsinization, collected by centrifugation and washed in PBS. eCTCs suspensions were incubated for 30 min with phycoerythrin (PE)-conjugated anti-CXCR4 antibody, washed, and then incubated for 1 h with anti-PE MicroBeads. After washing, cells were separated on LD (+) columns for positive and negative selection respectively, according to the manufacturer’s instructions. CXCR4-expression of sorted fractions was checked by FACS-analysis with the PE-conjugated sorting antibody. In addition, total eCTCs, as well as sorted fractions, were analyzed by FACS for CK20 and CD133 expression.

#### SCID xenografts

**Fig. 2:**
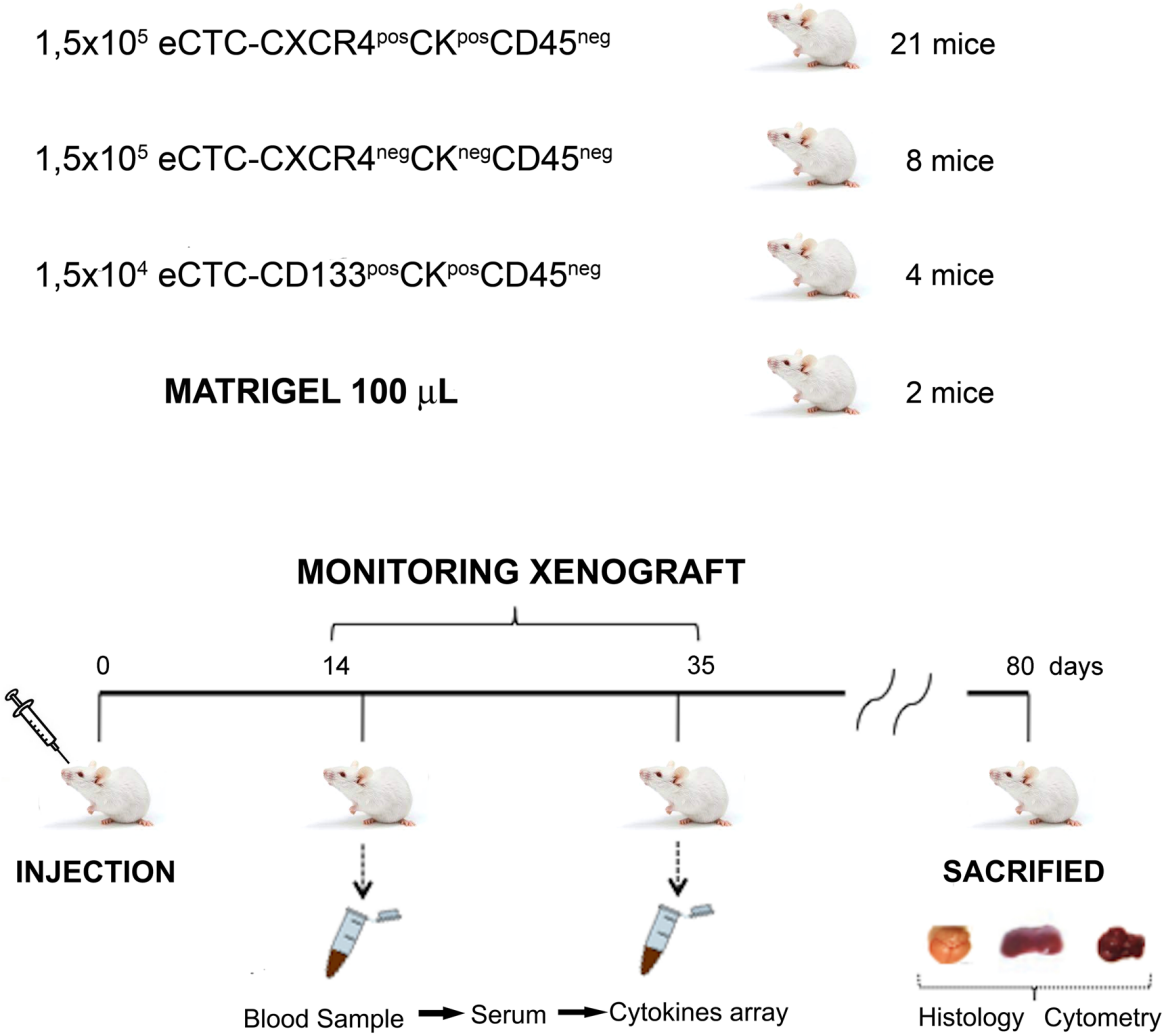
Animal experiment design

Severe combined immunodeficiency (SCID) mice (Harlan Laboratories) were cared for according to the institutional guidelines for animal care. The eCTCs were subcutaneously injected, without anaesthesia, in the mice right flank. 1.5 × 10^5^ tumour cells eCTCs-CXCR4^pos^CK20^pos^CD45^neg^ were suspended in 100 μl of matrigel (BD Biosciences) and injected in 21 mice. Moreover, eight mice were injected with 1.5 × 10^5^ tumour cells eCTCs-CXCR4^neg^CK20^neg^CD45^neg^, four mice with 1 × 10^4^ tumour cells eCTCs-CD133^pos^CK20^pos^CD45^neg^ and two with matrigel alone, for a total of 35 mice (Fig. 2). Two and 5 weeks after injection, whole blood was collected from tail vein and correspondent serum was analysed with the Human Cytokine Array kit. At day 80, organs and tissues were collected by scarified animals. Tissues were frozen and then sectioned (8 μm thick) on slides for immunofluorescence. Finally, the tissues were processed into cell suspensions for cytometric analysis. Tumor tissues excised from the 4 mice injected with 1 × 10^4^ tumour cells eCTCs-CD133^pos^CK20^pos^CD45^neg^ were analyzed for CD133 expression by immunohistochemistry (CD133-biotin Miltenyi Biotec) as previously described [14].

### Statistical analysis

Data are shown as means ± standard deviation (SD) of five blood samples (5 ml) collected from the patients. Statistical analyses and Kaplan Mayer curves were performed using MedCalc software, version 12.3. Three way analysis of variance (ANOVA) was used to analyse the variation of the CK20 and Fibronectin expression associated with tumour’s stage and with the cultivation time (7 and 14 days). Significance was set at p < 0.05.

## Results and discussion

### Collection and enrichment of CTCs

All patients were diagnosed with adenocarcinomas with moderately or poor differentiated histological grade. Five patients were in stages IV and III correspondent to stage (D) by Dukes classification. The correspondent mean value of the CTCs number investigated in whole blood was of 5 ± 1 cells/ml for CD133^pos^CD45^neg^ CTCs and 29 ± 3 cells/ml for CK20^pos^CD45^neg^ CTCs. The mean value of the CTCs number investigated in whole blood of two patients in stage II correspondent to stage (A) by Dukes, was of 2 ± 1 cells/ml for CD133^pos^CD45^neg^ and 1 ± 1 cells/ml for CK20^pos^CD45^neg^ CTCs. Further details about the clinicopathological features of the patients can be found in Table 1.

**Table 1:** Characteristics of patients involved in the study

Patient’s characteristics							
Patients	Stage	Grade	Tumour site	Sex	Age	OS (months)	DFS (days)
P1	IV (D)	Moderate	Right colon	M	73	10	90
P2	IV (D)	Poor	Para-colic	F	66	11	75
P3	III (D)	Moderate	Sigmoid	M	34	22	78
P4	II (A)	Moderate	Right colon	M	58	35	180
P5	II (A)	Moderate	Right colon	M	53	38	225
P6	IV (D)	Moderate	Sigmoid	F	76	9	100
P7	IV (D)	Poor	Right colon	M	65	11.5	97

*OS* overall survival, *DFS* disease free survival

**Fig. 3:**
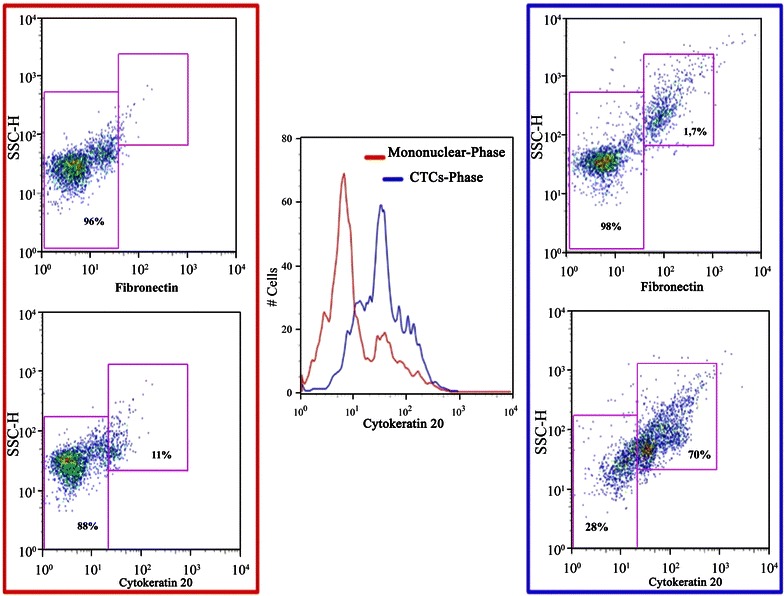
CTCs collection and enrichment phase. The figure reports CK20 (*down dot plots*) and Fibronectin (*up dot plots*) expression (*x-axis*, logarithmic scale) on side scatter-height (SSC-H, *y-axis*, logarithmic scale) collected from two different gradient density phases corresponding to the mononuclear cell layer (*red line*) and CTC phase (*blue line*) respectively. Central histogram shows the overlay CK20 expression (*x-axis*, logarithmic scale) on CTCs (*y-axis*, percentage) of the two different density ranges

This phase of research is focused to prove that the most of CTCs are included in the working density phase at 1.080 ÷ 1.090 (g/ml) [11]. Simulation experiment with a modified colon cancer cell line, GFP-HCT-116 cells, were performed. Cytometric analysis counted 88 ± 11 % of GFP-HCT-116 cells in the working density phase. Moreover, we investigated if the working density phase could be considered as the real CTCs enriched phase, also for colon cancer cells with epithelial to mesenchymal transition (EMT) phenotype. The presence of CTC expressing CK20, epithelial marker, and for fibronectin as mesenchymal markers, were evaluated, as reported in previous work [5]. Some reports suggest the migration of CTCs to the peripheral blood mononuclear cells layer during centrifugation [15]. The CTCs population was evaluated in both working density phase and mononuclear cells layer. The isolation of CK20 positive cells isolated from the mononuclear cell layer (red line) and from the working density phase (blue line) respectively, was resume in the Fig. 3 (central graphic). In particular, in advanced colon cancer (Dukes D stage), the percentage of cells positive for Fibronectin (up in the blue square of Fig. 3) was detected only in the working density phase (1.7 %). Conversely, CK20 positivity was detected in both mononuclear cell layer (11 %; Fig. 3, down in the red square) and in working density phase (70 %; Fig. 3, down in the blue square). These results confirmed that the working density phase represent the CTCs-enriched phase in Ficoll-gradient.

The CTCs detected in the working density phase of affected patients in Dukes D stage was 11 ± 1 % for the expression of CD133^pos^CD45^neg^ CTCs and 89 ± 2 % for CK20^pos^CD45^neg^ CTCs, while in Dukes A stage was 7 ± 1 % for CD133^pos^CD45^neg^ CTCs and of 3 ± 1 % for CK20^pos^CD45^neg^ CTCs.

### CTCs in vitro expansion and characterization

#### Phenotypic characterization

**Fig. 4:**
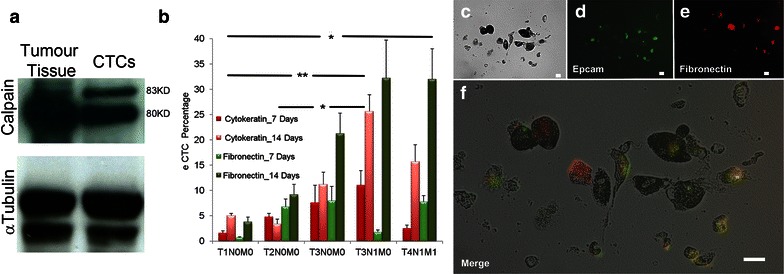
Phenotypic characterization of eCTCs. **a** Calpain expression was compared in the same patient between CTCs (collected before surgical treatment) and correspondent tumour tissue sample collected during surgical resection. Control α-tubulin immunoblot confirmed equal loading. **b** *Bar graph* displayed eCTCs dynamic phenotypic changes in different stage of tumour (according to Tumor-Node-Metastasis, UICC-2009) (data are presented as mean ± standard deviation). Multiple comparison procedures pairwise showed significant differences between advanced (T3N1M0, T4N1M1) versus localized colon cancer stages (T1N0M0, T2N0M0). *p < 0.05, **p ≤ 0.01. **c**–**f**, immunofluorescence characterization of eCTCs for epithelial (Epcam, *green channel*) and mesenchymal (Fibronectin, *red channel*) antigens expressions. Their co-expression on cell’s surface revealed the EMT phenotype (merged color image). *Scale bars* 50 μm

When induced to grow in vitro, tumor cells appeared as an adherent monolayer with or without sphere formation. The cultivation time was set at 14 days, after that the starting phenotypic configuration (analyzed just before CTCs seeding) changed in a percentage >35 %, as previously reported [11]. Non-adherent eCTCs were further expanded in suspension culture system to induce sphere formation. The spheres were analyzed for the expression of CD133, CK20 and CD45. The cancer cells forming spheres expressed CD133 (70 ± 8 %), CK20 (11 ± 9 %) and were CD45 negative. The characterization of eCTCs revealed that during their cultivation in vitro their phenotype changes. The composition of eCTCs population is characterized by different proportion of eCTCs subsets, according to the expression of epithelial antigens (CK20 or Epcam) or mesenchymal antigens as fibronectin (Fig. 4b). Three-way analysis of variance showed a significant difference for the expression of CK20 and fibronectin (p = 0.002) between two time-cultivation points (7 and 14 days in vitro). Moreover, the eCTCs phenotype showed significant difference in relation to tumour’s stage (Fig. 4b). Immunofluorescence analysis (Fig. 4c) showed the eCTCs phenotype expressing Epcam (green channel) and fibronectin (red channel); merged color image reported cellular elements characterized by the co-expression of fibronectin and epithelial antigens highlighting EMT phenotypes. We concluded that eCTCs displaying EMT phenotype are detectable both in localized and advanced colon cancer cases, with a prevalence in the advanced cases.

#### Cell cycle analysis

**Fig. 5:**
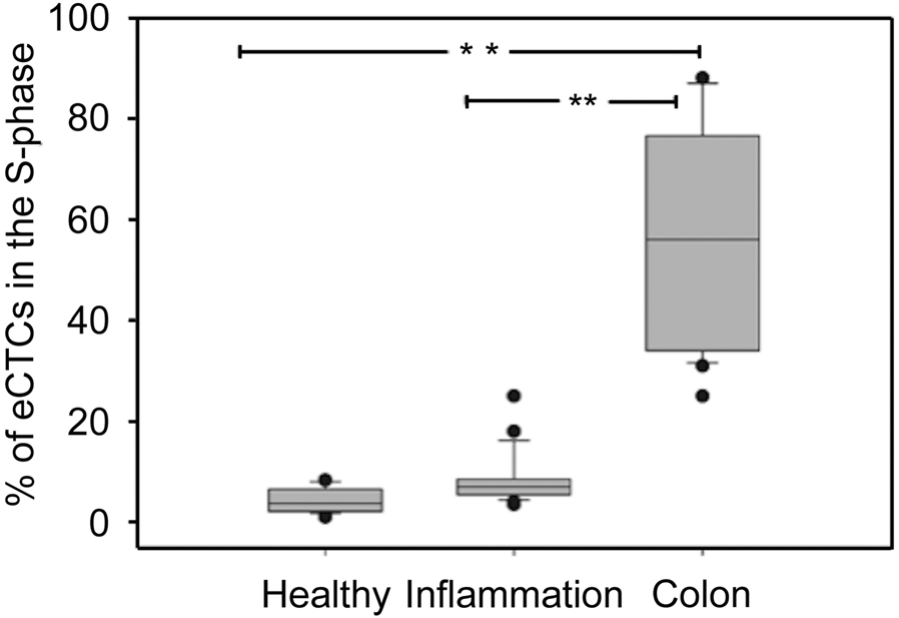
Cell cycle analysis. The *box plot* compares S-phase percentage of adherent cells (isolated in correspondence to the working density phase and briefly expanded according to the protocol here reported for CTCs) isolated from healthy volunteers, patients affected by chronic inflammation (ulcerative colitis and Crohn’s disease) and colon cancer.**p < 0.001

Our method tests in vitro, through the short-time cultivation, the intrinsic property of cancer cells: their independent and anarchical proliferation. In order to evaluate the proliferation rate of CTCs, we applied the same procedure of CTCs isolation and cultivation on blood samples collected by healthy volunteers, patients affected by chronic inflammation (ulcerative colitis and Crohn’s disease) and cancer colon patients. Twenty-nine subjects (14 males and 15 females) with an age ranging from 25 to 77 years, were enrolled. The cell suspensions isolated from healthy blood samples, in correspondence of working density phase, contained epithelial cells not able to expand in vitro. In these adherent cells, the mean value of S-phase percentage was of 4.4 ± 2.3 %. Analogue adherent cells, with limited proliferation, were observed in subjects affected by chronic inflammation, and their S-phase was of 8 ± 5 %. Finally, the eCTCs isolated from samples of colon cancer patients were able to proliferate for at least two passages with a S-phase of 57 ± 20 %. The comparison of S-phase between healthy and inflammation versus colon cancer cases was statistically significant (p < 0.001; Fig. 5). The experiment proved that it is possible to find circulating cells with an epithelial phenotype in subjects unaffected by cancer disease, so the count of CTCs can be not sufficient to specifically define cancer condition. In fact, the sample of healthy cells isolated in vitro was unable to survive and expand while the sample of tumor cells was able to survive and expand as proved by S-phase percentage.

#### Cytokines array

**Fig. 6:**
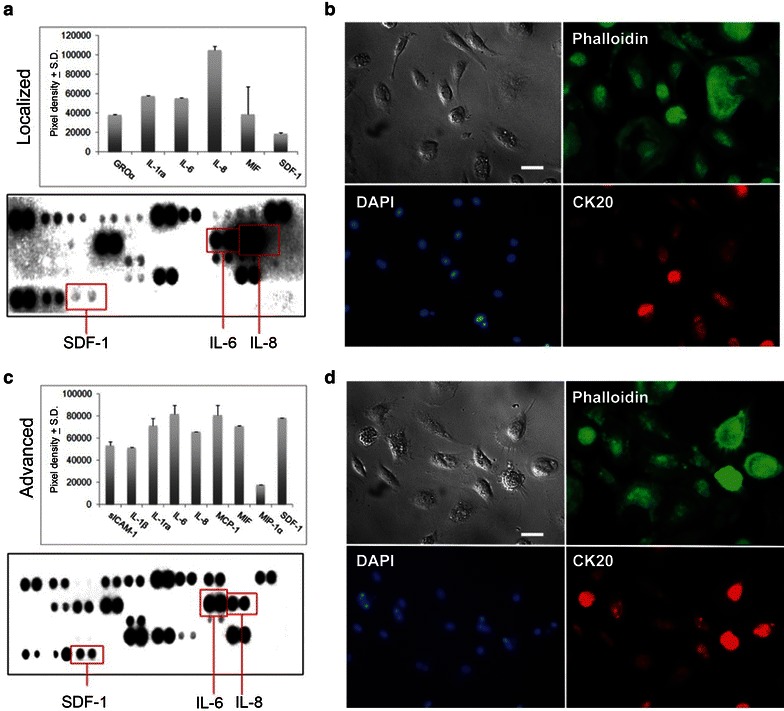
Cytokines array and morphology of adherent eCTCs. Cytokines concentration was analysed in collected conditioned medium of eCTCs aged 14 days. Histograms (**a**) and (**c**) show mean spot pixel density from the array membranes (*red boxes*) by localized and advanced cases, respectively (data are presented as mean ± standard deviation). In all conditioned medium, detectable levels of IL-6, IL-8, and SDF-1 cytokines were found. The histograms are representative of at least three independent experiments. The array membranes pictures show one representative case. Morphology of eCTCs in adhesion isolated by localized (**b**) and advanced (**d**) cases is shown; in particular, brightfield image, phalloidin (*green* staining of cytoplasm), DAPI (*blue* staining of nuclei) and CK20 (*red* staining of positive cells). *Scale bars* 50 μm

Moreover, we analyzed the products released by the eCTCs derived from localized and advanced colon cancer cases. The corresponding amount of cytokines detected in conditioned medium are reported in Fig. 6a, c. The membranes show a production of IL-6 and IL-8 cytokines with different concentration between the medium derived by localized and advanced colon cancer cases. Moreover, we focused our attention on the production of SDF-1 (stromal cell-derived factor-1). The regulatory system SDF-1/CXCR4 (Chemokine (C-X-C motif) receptor 4) is involved in the metastasis phase of colon, lung, and breast cancers [16–18]. CXCR4 is an antigen key regulator of tumor invasiveness leading to local progression and tumor metastasis [19]. We found levels of SDF-1 in the conditioned medium by eCTCs of both localized 2/2 cases with a mean value of pixel density of [(17 ± 20) × 10^3^] and advanced 5/5 cases with a mean value of pixel density of ((75 ± 20) × 10^3^) cancer cases, as shown in Fig. 6a, c respectively. We concluded that SDF-1 is released by eCTCs derived from both localized and advanced colon cancer cases. Moreover, the evidence of the production ex vivo of SDF-1 was used as tool to monitor cancer cell survival in mice that during xenograft experiment did not develop macroscopic lesions. Clinical cases showing primary cultures of adherent eCTCs for localized (Fig. 6b) and advanced cases (Fig. 6d) were reported.

### SCID xenografts

#### Characterization of injected circulating cancer cell

**Fig. 7:**
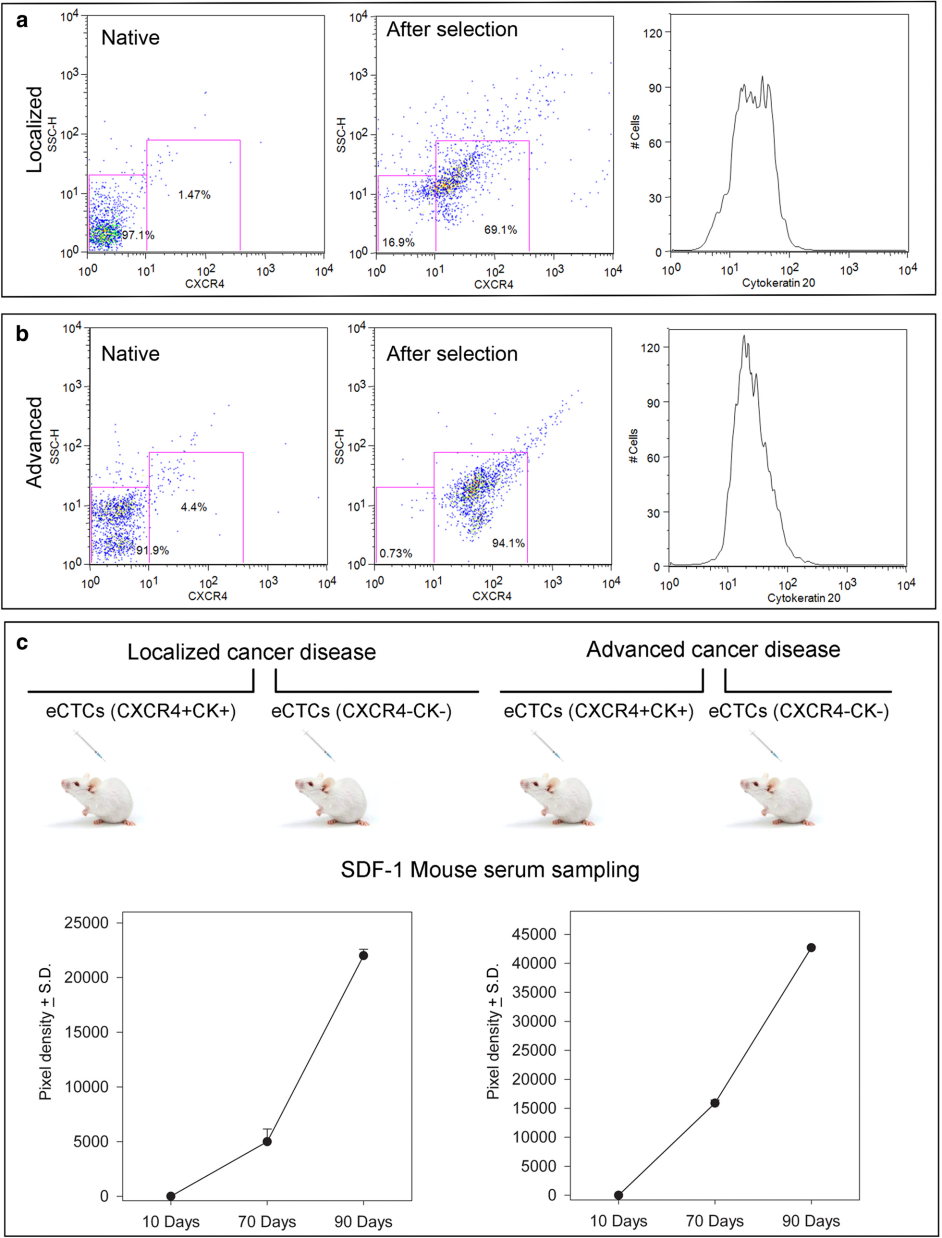
Characterization of injected circulating cancer cells and survival trend. Representative *dot plots* show cell surface CXCR4 expression (*x-axis*) versus side scatter (SSC; *y-axis*) of pre-sorting (*naive*) and post-sort tumour cellular suspensions (**a**, **b**). After cell sorting the post-sorting cell population was evaluated for the expression of CK20 reported in histograms (*right panels*) respectively in localized (**a**) and advanced (**b**) colon cancer. **c** Trends of SDF-1 (data are represented as mean ± standard deviation) in serum of mice injected with eCTCs-CXCR4^pos^CK^pos^ segment were reported. In mice injected with eCTCs-CXCR4^pos^CK^pos^ derived from localized colon case the levels of serum SDF-1 shows a sharp increase which peaking at days ninety (*left panel*). In mice injected with eCTCs-CXCR4^pos^CK^pos^ derived from advanced colon case the levels of serum SDF-1 show a steeper trend of the curve (*right panel*)

On the basis of SDF-1 production by eCTCs, CXCR4 expression was investigated. The populations of eCTCs-CXCR4^pos^ were 9.0 ± 2.8 and 22.2 ± 5.4 % in localized and advanced cases, respectively. Moreover, adherent eCTCs were sorted for CXCR4 expression and two different populations were distinguished: eCTCs-CXCR4^neg^ and eCTCs-CXCR4^pos^ (Fig. 7a, b). eCTCs-CXCR4^pos^ sorted after 14 days of cultivation were further analyzed for CK20 expression. The mean percentage of eCTCs-CXCR4^pos^CK20^pos^CD45^neg^ was 77 ± 3 % in localized (Fig. 7a, right) and 81 ± 9 % in the advanced cases (Fig. 7b, right). We injected eCTCs-CXCR4^pos^CK20^pos^CD45^neg^ in SCID mice (Fig. 7c, up). The group of mice injected with eCTCs-CXCR4^pos^CK20^pos^CD45^neg^ segment did not develop macroscopic evidences of tumor lesions. In order to verify the success of transplantation, we used SDF-1 released by eCTCs as guideline for monitoring cancer cells survival trend (Fig. 7c, down). The levels of SDF-1 were detectable in serum of the 80 % of mice injected, with a different concentration in localized and advanced cases (Fig. 7c, down). Conversely, SDF-1 was not detectable in mice injected with selected eCTCs-CXCR4^neg^CK20^neg^CD45^neg^ segment, suggesting that these cells were unable to survive in vivo.

**Fig. 8:**
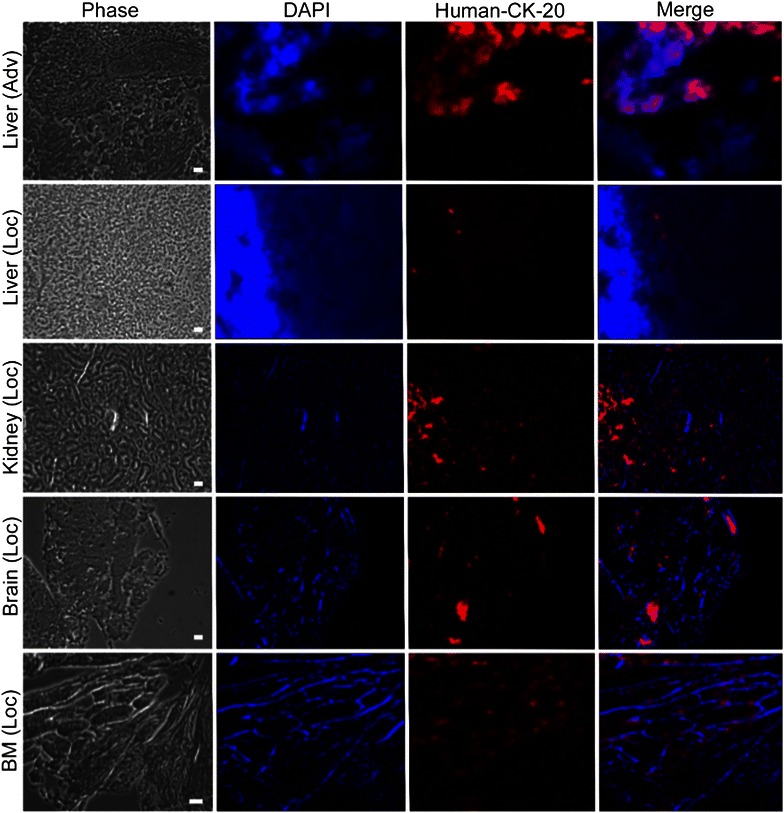
Xenograft developed with circulating tumor cells (eCTCs-CXCR4^pos^CK20^pos^). Immunofluorescence analysis was performed using human monoclonal antibody direct against CK20 antigen. Images of liver derived from mice injected with eCTCs-CXCR4^pos^CK^pos^ segment isolated by localized (Loc) and advanced (Adv) colon cases, are reported. Moreover, immunofluorescence analysis for kidney, brain and bone marrow (BM) collected from mice injected with eCTCs-CXCR4^pos^CK^pos^ segment derived from localized colon cancer case, is shown. Bright field image, DAPI (*blue*), CK20 (*red*) and merge are shown. *Scale bars* 100 μm

Finally, immunofluorescence analysis on mice tissues for the expression of CK20 confirmed the ability of the eCTCs-CXCR4^pos^CK20^pos^ segment to survive and disseminate in the mice, even if derived from localized colon cancer patients. The histological sections of liver, brain, kidney and bone marrow mice show human eCTCs (derived from localized colon cancer cases) organized in microcolonies, without deep alteration of tissue texture (Fig. 8). Conversely, the sections of mice livers treated with eCTCs-CXCR4^pos^CK20^pos^ (derived from advanced colon cancer cases), show an invasion of eCTCs, organized in clusters, subverting original tissue architecture. Moreover, the cytometric analysis of tissue suspension derived from mice liver show 27 ± 14 % CK20^pos^ cells in localized and of 45 ± 20 % CK20^pos^ cells in advanced cases (Additional file 1: Figure S3).

In conclusion, eCTCs-CXCR4^pos^CK20^pos^ segment, detectable in different proportion in localized and advanced cases, is able to survive and disseminate in tissues other than their primary localization.

#### Characterization of injected circulating cancer stem cell

Non-adherent forming sphere, eCTCs-CD133^pos^CK20^pos^CD45^neg^, derived from localized and advanced colon cancer cases, were used to perform xenografts. Within 2 weeks, the 100 % of mice presented macroscopic lesions on the right flank, in correspondence of site of CTCs injections (Additional file 1: Figure S4), confirming the intrinsic tumorigenic property of this eCTCs subset.

Overall, the experiments demonstrated that the eCTCs-CXCR4^pos^CK20^pos^ CTCs segment is not tumorigenic but able to disseminate and survive in animal tissues. In the literature, the positivity for CXCR4 and CD133 was used in metastatic case to detect cancer stem cells (CSCs). The migration of invasive CSCs is primarily mediated by CXCR4/SDF-1 axis gradient, able to attract tumor cells regulating them for proliferation and invasion [20, 21]. Consequently, on the basis of our results, in early colon cancer cases it is possible to detect CTCs expressing CXCR4. This CTCs subset correlated with a dissemination potential. Moreover, the CTCs expressing CD133, like the CSCs, represent the subset with tumorigenic power. Our in vitro and in vivo characterization of eCTCs expressing CD133 revealed that their plasticity is a dynamic property increasing with the stage of the disease [22].

### Benchmarking clinical data and metastatic risk assessment

**Fig. 9:**
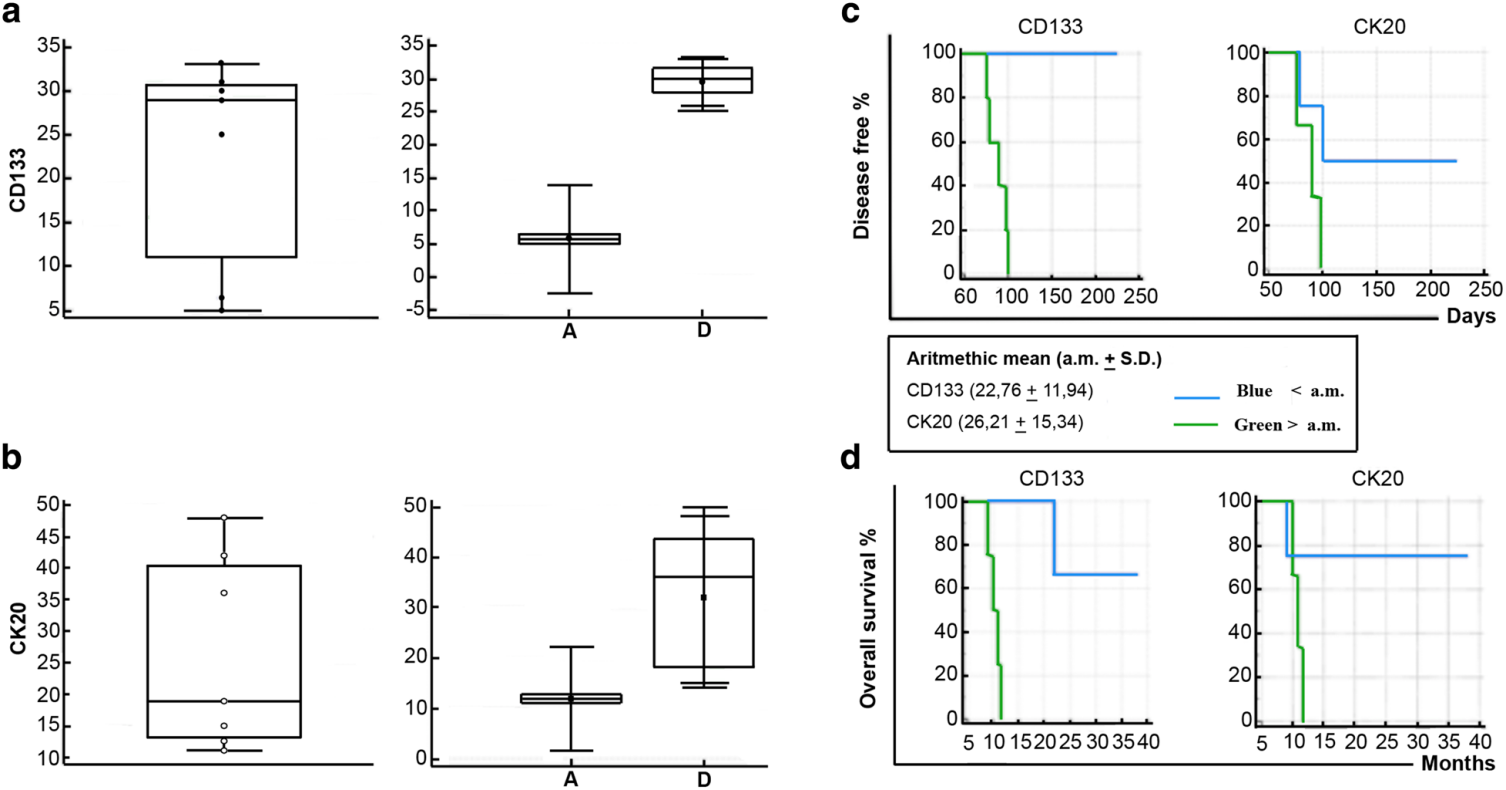
Benchmarking clinical data. In **a** the *box* of CD133 and in **b** CK20 positivity distribution applying Kolmogorov–Smirnov test. Correspondent arithmetic means for each variable was used as cut-off value to stratify the patients according to low (**a** *box*) and high (**d** *box*) expression for CD133 and CK20 antigens, respectively. Moreover, arithmetic means for each variable was used as cut-off value to distinguish two subgroups of patients. In function of the low and high expression for each antigen the relative overall survival (**c**) and disease free (**d**) were calculated

The observation of the patients’ outcomes was conducted for 3 years on a limited panel of seven patients. The small number of patients enrolled in this study have not statistical significance, but the trend curves resulting suggestive. In particular, the arithmetic mean of the expression value for CD133 and CK20 of CTCs was used as cut-off to stratify the patients. The curve of overall survival (OS) shows an adverse prognosis for the patients with high CD133^pos^-CTCs. These cells are also tumorigenic in the mice model. In terms of disease free (DF), the patient with high percentage of CTCs expressing CK20 have adverse prognosis (Fig. 9). This subset corresponds to the pool of eCTCs able to survive and disseminate in the mice model. Tumor tissue remodelling during cancer progression unbalances the proportion of all cancer cells subpopulations, as reflected in the heterogeneous composition of circulating progeny. To better monitor the patient clinical course it should be necessary to know the different subsets of cancer cells and their dynamic changes. Our study highlights that tumorigenic subset (CD133^pos^) represents the predominant subtype of CTCs in the last phase of tumour progression. On the other hand, the non-tumorigenic circulating progeny (CK20^pos^), prevalently detected in the localized phase of disease, is able to disseminate.

Despite the low sample size, these results suggest that the high presence of non-tumorigenic CTCs subset can negatively influence the disease free survival, probably promoting relapses in the tissue were they are disseminated.

## Conclusion

Detailing CTCs in distinct subsets, defined by qualitative/quantitative measurement, might be useful to stratify the patients according to a personalized metastatic risk score. The “emo-cytometric” approach on CTCs here described can be considered an interesting tool to improve the clinical surveillance and reducing the clinical cost.

## Additional file

10.1186/s12967-016-0876-y Sensibility, specificity and purity of CTCs detection methodology. The sensitivity of the methodology was calculated through the formula employing mean values (expressed in percentage) for each CTCs subsets identified by the combined expression of CK20 and CD45, found in the total cellular suspension collected from the working density phase. The sensitivity or the capability to detect the real subset of CTCs CK20^pos^ corresponded to 91 %. The specificity, corresponding to the probability of a negative test, was calculated at about 87 %. Finally, the purity was at 75 %.** Figure S2.** Resolution of CTCs detection methodology. To verify that the collected fraction was enriched in cancer cells, HCT 116 cells were infected with pAdenoVator-CMV-IRES-GFP reporter. Human cancer colon cell lines HCT 116 were cultured in RPMI1640 medium containing 10 % fetal bovine serum (FBS), 2 mmol/l L-glutamine, and 30 mg penicillin G/0.05 g streptomycin. Cells were plated at 8 x 10^6^ per well onto a six-well plate 24 hours before infection, and were infected with adenoviral vector. In order to perform infections, HCT 116 cells were incubated with pAdenoVator-CMV5(CuO)-IRES-GFP (Qbiogene, Carlsbad, CA) in serum free medium for 1 hour at 37 °C. Both vectors were used at multiplicity of infection (m.o.i.) of 3000 physical particles/cell, experimentally determined as the lowest m.o.i. at which the majority of the cell population is infected (as assessed by EGFP expression). Twenty-four hours later, both adherent and floating cells were harvested, washed in PBS and counted. Different concentration of HCT 116-GFP (HCT 116*) were put in entire blood sample (5 ml) and were evaluated through cytometric analysis. The resolution for the minimal concentration of HCT 116* (8 x 10^3^ cell/5 ml) put in a volume of peripheral blood sample of 5ml, useful to detect them in the working density phase, was calculated at 5,8 cells/5 ml (B).** Figure S3. **DTCs in livers of mice treated with localized and advanced cancer eCTCs. Dot Plots report the expression of CK20 antigen on human colon cancer cells disseminated within liver tissue of mouse submitted to xenograft procedure. In particular, dot plot in (A) shows human colon cancer cell CK20 positive founded in liver tissue of mouse injected with eCTCs-CXCR4^neg^CK^neg^ referred as control. Dot plots in (B) and (C) show human cancer colon cells expressing CK20 marker in liver tissues of mouse injected with eCTCs-CXCR4^pos^CK^pos^ derived from localized (B) and advanced (C) colon cancer cases respectively.** Figure S4.** xenograft developed with circulating stem cells. Xenograft procedure developed injecting eCTCs-CD45^neg^CD133^pos^ organized in spheres (A). In (B) immunofluorescence positive for CD133 (green staining). In (C) Tumour formations produced within 2 weeks and after 80 days. Immunohistochemical analysis shows the distribution of the cancer colon cells expressing CD133 (brown staining) in the tumour sections of 8 μm (D).

## Abbreviations

CTCs: circulating tumor cells
SCID: severe combined immune deficiency
CXCR4: chemokine (C-X-C motif) receptor 4
CK20: cytokeratin 20
DTCs: disseminated tumor cells
TNM: tumor-node-metastasis
PE: phycoerythrin
eCTC: expanded circulating tumor cells
PBS: phosphate-buffered saline
FCS: fetal calf serum
EMT: epithelial-mesenchymal transition
CSC: cancer stem cell
