# Supplementary material for: Ex-vivo characterization of circulating colon cancer cells distinguished in stem and differentiated subset provides useful biomarker for personalized metastatic risk assessment
Source: J Transl Med. 2016 May 12;14:133. doi: 10.1186/s12967-016-0876-y (PMC4866436; doi:10.1186/s12967-016-0876-y)

**Figure S1. Sensibility, specificity and purity of CTCs detection methodology.**

The sensitivity of the methodology was calculated through the formula employing mean values (expressed in percentage) for each CTCs subsets identified by the combined expression of CK20 and CD45, found in the total cellular suspension collected from the working density phase. The sensitivity or the capability to detect the real subset of CTCs CK20^pos^ corresponded to 91%. The specificity, corresponding to the probability of a negative test, was calculated at about 87%. Finally, the purity was at 75%.

**
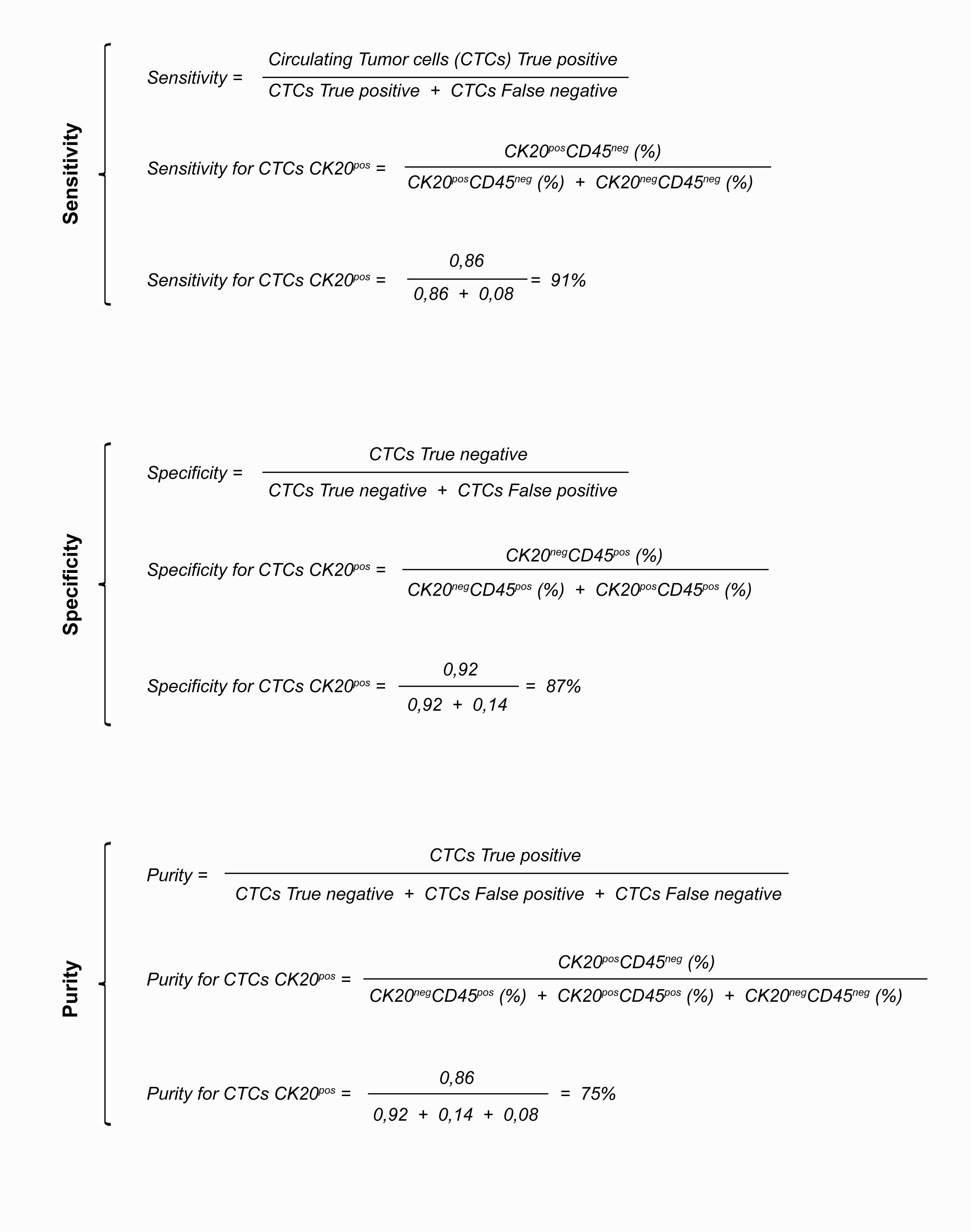
**

**Figure S2. Resolution of CTCs detection methodology**

To verify that the collected fraction was enriched in cancer cells, HCT 116 cells were infected with pAdenoVator-CMV-IRES-GFP reporter. Human cancer colon cell lines HCT 116 were cultured in RPMI1640 medium containing 10% fetal bovine serum (FBS), 2 mmol/l L-glutamine, and 30 mg penicillin G/0.05 g streptomycin. Cells were plated at 1X10^6^ per well onto a six-well plate 24 hours before infection, and were infected with adenoviral vector. In order to perform infections, HCT 116 cells were incubated with pAdenoVator-CMV5(CuO)-IRES-GFP (Qbiogene, Carlsbad, CA) in serum free medium for 1 hour at 37 °C. Both vectors were used at multiplicity of infection (m.o.i.) of 3000 physical particles/cell, experimentally determined as the lowest m.o.i. at which the majority of the cell population is infected (as assessed by EGFP expression). Twenty-four hours later, both adherent and floating cells were harvested, washed in PBS and counted. Different concentration of HCT 116-GFP (HCT 116*) were put in entire blood sample (5 ml) and were evaluated through cytometric analysis. The resolution for the minimal concentration of HCT 116* (8 x 10^3^ cell/5 ml) put in a volume of peripheral blood sample of 5ml, useful to detect them in the working density phase, was calculated at 5,8 cells/5 ml (B).


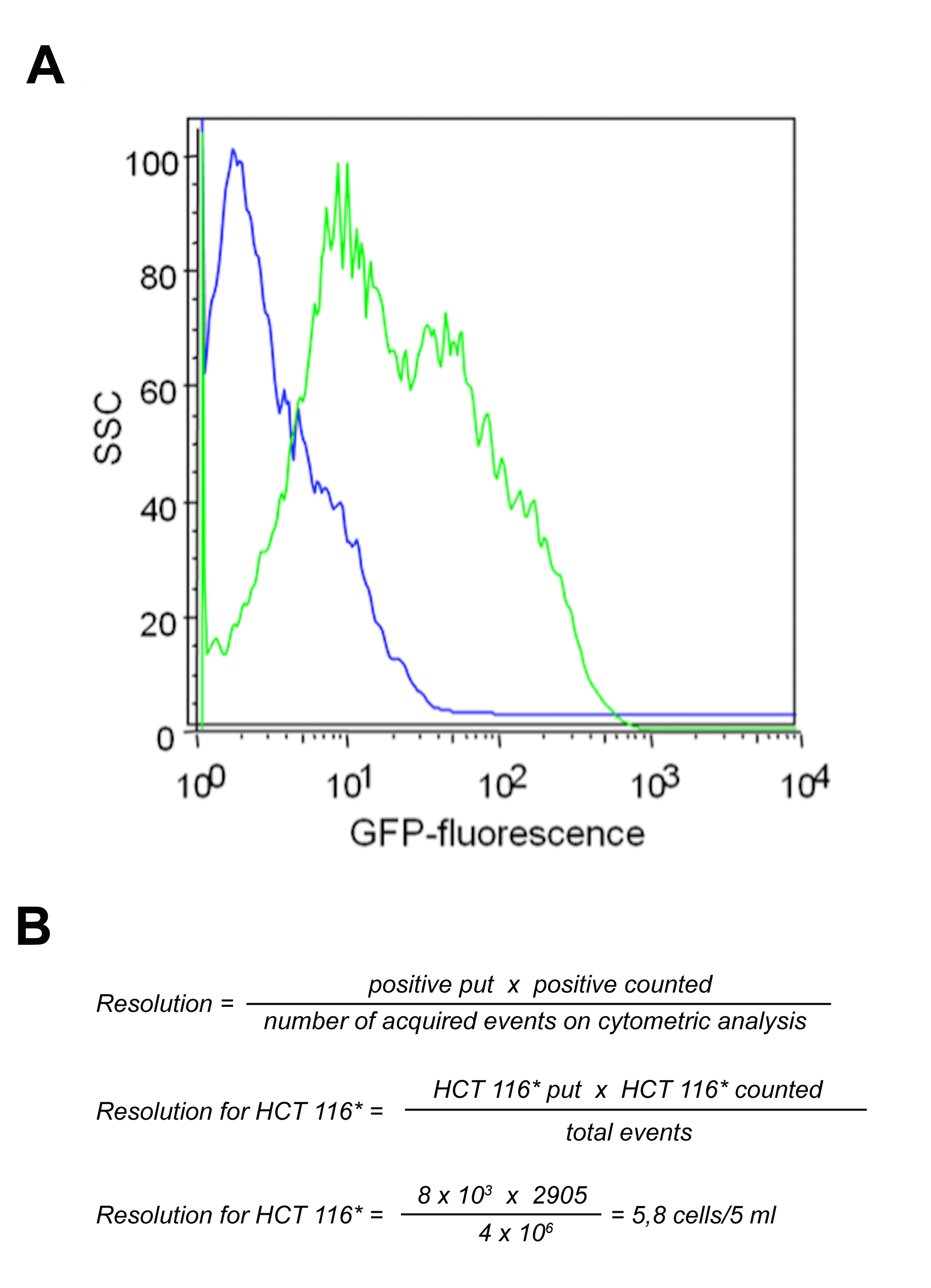


**Figure S3.** **Cytometric analysis of liver cell suspension for CK20.** Dot Plots report the expression of CK20 antigen on human colon cancer cells disseminated within liver tissue of mice submitted to xenograft procedure. In particular, dot plot in (A) shows human colon cancer cell CK20 positive founded in liver tissue of mice injected with eCTCs-CXCR4^neg^CK^neg^ as negative control. Dot plots in (B) and (C) show human cancer colon cells expressing CK20 in liver tissues of mice injected with eCTCs-CXCR4^pos^CK^pos^ derived from localized (B) and advanced (C) colon cancer cases, respectively.


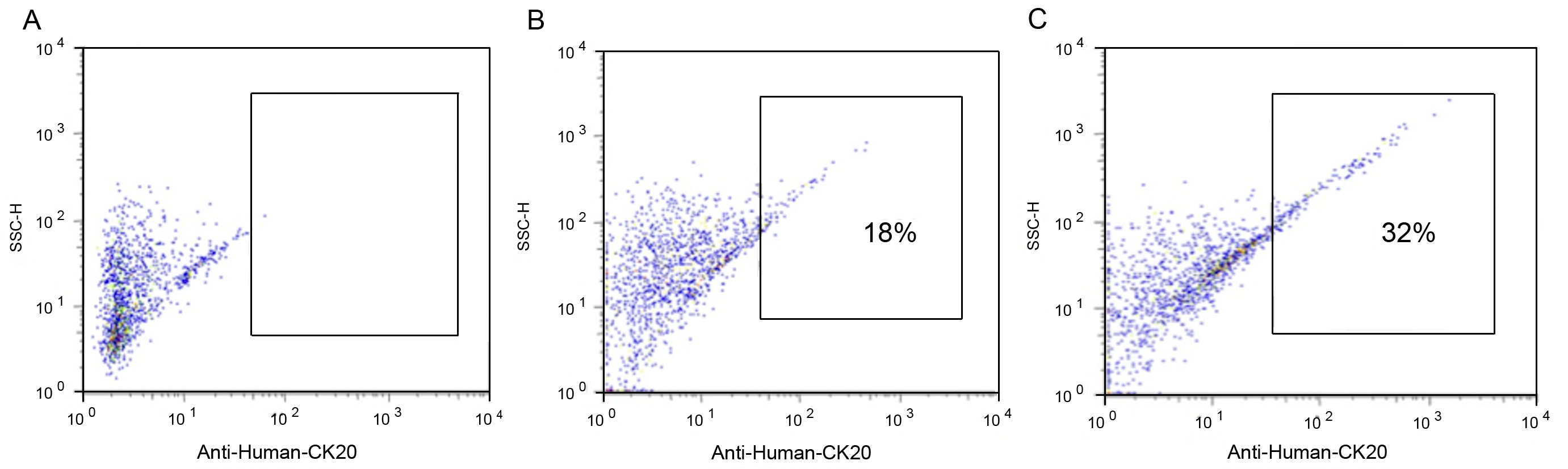


**Figure S4. Xenograft developed with circulating tumor stem cells (eCTCs-CD133^pos^CK20^pos^).** Xenograft procedure developed injecting eCTCs-CD133^pos^CK20^pos^ organized in spheres (A). In (B), immunofluorescence positivity for CD133 (green staining). In (C), tumour lesions produced within 2 weeks and after 80 days. At 80 days, immunohistochemical analysis shows the distribution of CD133 expression (brown staining) in sections of tumor lesion (8 μm; D).


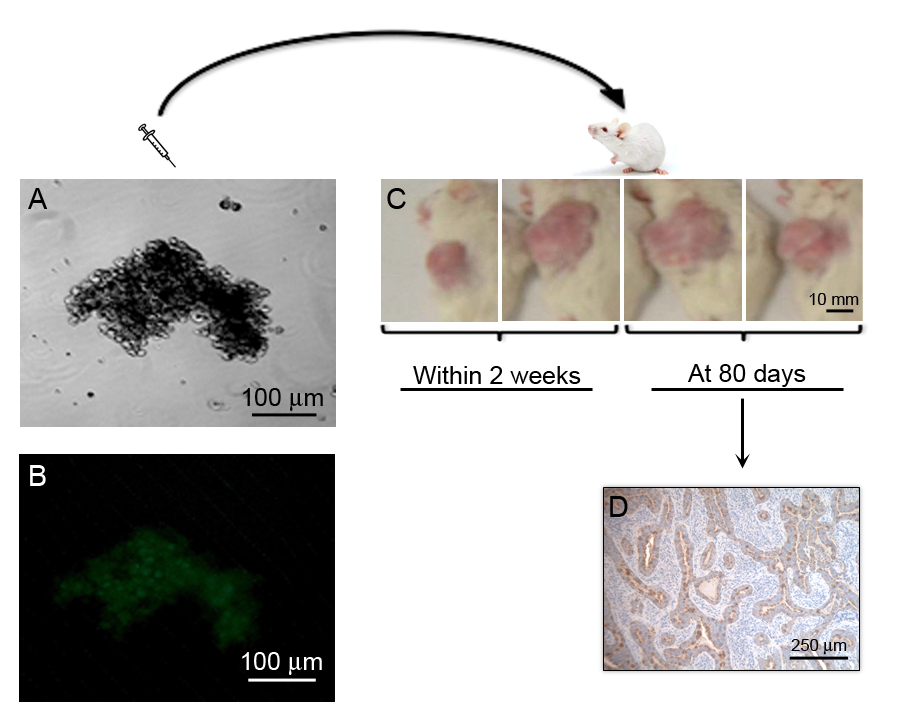

Supplement: Supplementary file 1 — 10.1186/s12967-016-0876-y Sensibility, specificity and purity of CTCs detection methodology. The sensitivity of the methodology was calculated through the formula employing mean values (expressed in percentage) for each CTCs subsets identified by the combined expression of CK20 and CD45, found in the total cellular suspension collected from the working density phase. The sensitivity or the capability to detect the real subset of CTCs CK20pos corresponded to 91 %. The specificity, corresponding to the probability of a negative test, was calculated at about 87 %. Finally, the purity was at 75 %. Figure S2. Resolution of CTCs detection methodology. To verify that the collected fraction was enriched in cancer cells, HCT 116 cells were infected with pAdenoVator-CMV-IRES-GFP reporter. Human cancer colon cell lines HCT 116 were cultured in RPMI1640 medium containing 10 % fetal bovine serum (FBS), 2 mmol/l L-glutamine, and 30 mg penicillin G/0.05 g streptomycin. Cells were plated at 8 x 10^6^ per well onto a six-well plate 24 hours before infection, and were infected with adenoviral vector. In order to perform infections, HCT 116 cells were incubated with pAdenoVator-CMV5(CuO)-IRES-GFP (Qbiogene, Carlsbad, CA) in serum free medium for 1 hour at 37 °C. Both vectors were used at multiplicity of infection (m.o.i.) of 3000 physical particles/cell, experimentally determined as the lowest m.o.i. at which the majority of the cell population is infected (as assessed by EGFP expression). Twenty-four hours later, both adherent and floating cells were harvested, washed in PBS and counted. Different concentration of HCT 116-GFP (HCT 116*) were put in entire blood sample (5 ml) and were evaluated through cytometric analysis. The resolution for the minimal concentration of HCT 116* (8 x 10^3^ cell/5 ml) put in a volume of peripheral blood sample of 5ml, useful to detect them in the working density phase, was calculated at 5,8 cells/5 ml (B). Figure S3. DTCs in livers of mice treated with loc [file 12967_2016_876_MOESM1_ESM.docx]
